# Supplementary material for: Trans-Golgi protein TVP23B regulates host-microbe interactions via Paneth cell homeostasis and Goblet cell glycosylation
Source: Nat Commun. 2023 Jun 20;14:3652. doi: 10.1038/s41467-023-39398-1 (PMC10282085; doi:10.1038/s41467-023-39398-1)

Supplemental Methods

FITC-dextran permeability assay

Intestinal permeability was assessed by administration of FITC-dextran 4000 (Sigma). After a 4-hour fast, mice were orally gavaged with FITC-dextran (20 mg/100 g body weight). Whole blood was obtained from the submandibular plexus and fluorescence was measured in the serum by a fluorometer (BioTek) at 488 nm.

Lamina propria leukocyte isolation

Lymphocytes from small intestinal and colonial lamina propria were isolated as previously described with minor modification[1]. Briefly, the small intestine and colon were flushed with the ice-cold PBS with 2% FBS, opened longitudinally, and cut into 1 cm pieces. The tissues were incubated with 15 ml of PBS with 1 mM EDTA, 1 mM DTT, and 2% FBS at 37°C in a shaker at 250 rpm for 15 min. The tissues were washed in PBS with 2% FBS and placed in RPMI-1640 with 2% FBS, 50 µg/ml of DNase I, and 30 µg/ml of Liberase for small intestine or 50 µg/ml of Liberase for colon. After incubation at 37°C for 30 min in a shaker at 250 rpm, the solution was vortexed and passed through a cell strainer. The cell suspensions were washed and applied to a 40%:80% Percoll gradient. After centrifugation at 800g for 20 min, lymphocytes were collected from the gradient interface.

16S rRNA sequencing and data analysis on tissue and fecal sample

The hypervariable region V3 & V4 of bacterial 16S rRNA gene were captured using Zymo Quick 16S protocol (Zymo Quick-16S NGS Library Prep Kit (Catalog # D6400)). PCR product was cleaned using Agencourt AmpureXP beads from Beckman Counter Genomics. Illumina adapter and barcode sequences were ligated to amplicon in order to attach them to MiSeqDx flow cell and for multiplexing. Quality and quantity of each sequencing library was assessed using Bioanlyzer and picogreen measurements, respectively. About 6pM of pooled library was loaded onto a MiSeqDX flow cell and sequenced using PE300 (Paired end 300 bp) v3 kit. Raw fastq files were demultiplexed based on unique barcodes and assessed for quality. Samples with more than 50K QC pass sequencing reads were used for downstream 16S Operational Taxonomic Unit (OTU) analysis.

16S gene sequencing analysis pipeline

Taxonomic classification and OTUs abundance analysis was done using CLC Bio microbial genomics module version 12.0 (https://www.qiagenbioinformatics.com/plugins/clc-microbial-genomics-module/). Individual sample reads were annotated with Greengene database and taxonomic features were determined. Alpha and beta diversity analysis were done to measure the within- and between- sample diversity, respectively. Abundance data was used for numeric Principal Component Analysis (PCA) in SVS, Golden Helix Software. Raw fastq files from this study will be submitted to Sequence Read Archive and are also available on direct request.

In vivo NK Cell and CD8+ T cell cytotoxicity analyses.

For the CTL assay, splenocytes were harvested from B6 mice and divided in half. According to established methods, half were stained with 5 µM CFSE ($CFSE^{hi}$), and half were labeled with 0.5 µM CFSE ($CFSE^{lo}$). $CFSE^{hi}$ cells were loaded with SIINFEKL peptide(5 µM). $CFSE^{lo}$ cells were not stimulated. $CFSE^{hi}$ and $CFSE^{lo}$ cells were mixed (1:1) and 2×10^6 cells were administered to naïve mice and mice immunized with alum-ova through intravenous injection. Blood was collected 48 h after transfer, and CFSE intensities from each population were assessed by flow

cytometry. Lysis of target (CFSE^{hi}) cells was calculated as: % lysis = [1 – (ratio control mice/ratio vaccinated mice)] × 100; ratio = percent CFSE^{lo}/percent CFSE^{hi}. To measure NK cell-mediated killing, splenocytes from control C57BL/6J (0.5 μM Violet; Violet^{lo}) and B2m$^{-/-}$ mice (5 μM Violet; Violet^{hi}) were stained with CellTrace Violet. Equal numbers of Violet^{hi} and Violet^{lo} cells were transferred to recipient mice by retro-orbital injection. Twenty-four hours after transfer, blood was collected and Violet intensity from each population was assessed by flow cytometry. % lysis = [1 – (target cells/control cells) / (target cells/control cells in B2m$^{-/-}$)] × 100.

Flow cytometry

Peripheral blood cells were isolated and red blood cell (RBC) lysis buffer was added to remove RBCs. Peripheral blood cells were washed with FACS staining buffer (PBS with 1% (w/v) BSA) and then centrifuged at 500 × g for 5 minutes. Peripheral blood cells were stained for 1 hour at 4°C, in 100 μl of a 1:200 cocktail of fluorescence-conjugated antibodies to 8 cell surface markers encompassing the major immune lineages: B220, CD19, CD3ε, CD4, CD8α, CD11b, F4/80, NK 1.1 and 1:200 Fc block.

Lamina propria lymphocytes were stained at a 1:200 dilution in the presence of anti-mouse CD16/32 antibody for 30 min at 4°C with fluorochrome-conjugated antibodies against CD45, CD19, TCRβ, CD4, CD8α, MHC-II, CD11c and CD64. To analyze IL-17 production, lamina propria lymphocytes were stimulated with the Leukocyte Activation Cocktail, with BD GolgiPlug™ (BD) for 3 hours. For intracellular staining, cells were stained with surface markers and then fixed, permeabilized, and stained with antibodies specific for IL-17 or FOXP3.

ALDH activity was measured using an Aldefluor Assay kit (Stem Cell Tech) per manufacturers guidelines.

RNA sequencing library preparation

RNA was extracted using RNeasy Mini Kit (QIAGEN) according to the manufacturer’s protocol. RNA quantity and purity was assessed on a NanoDrop 2000 spectrophotometer (Thermo Fisher Scientific), and integrity was measured on an Agilent Bioanalyzer 2100 (Agilent Technologies). RNA-seq libraries were prepared with the KAPA Stranded RNA-Seq Kit with RiboErase (HMR) (KAPA Biosystems) according to the manufacturer’s protocol.

Supplemental References

1. Kim E, Tran M, Sun Y, Huh JR. Isolation and analyses of lamina propria lymphocytes from mouse intestines. *STAR Protoc* **3**, 101366 (2022).

Supplemental Figures

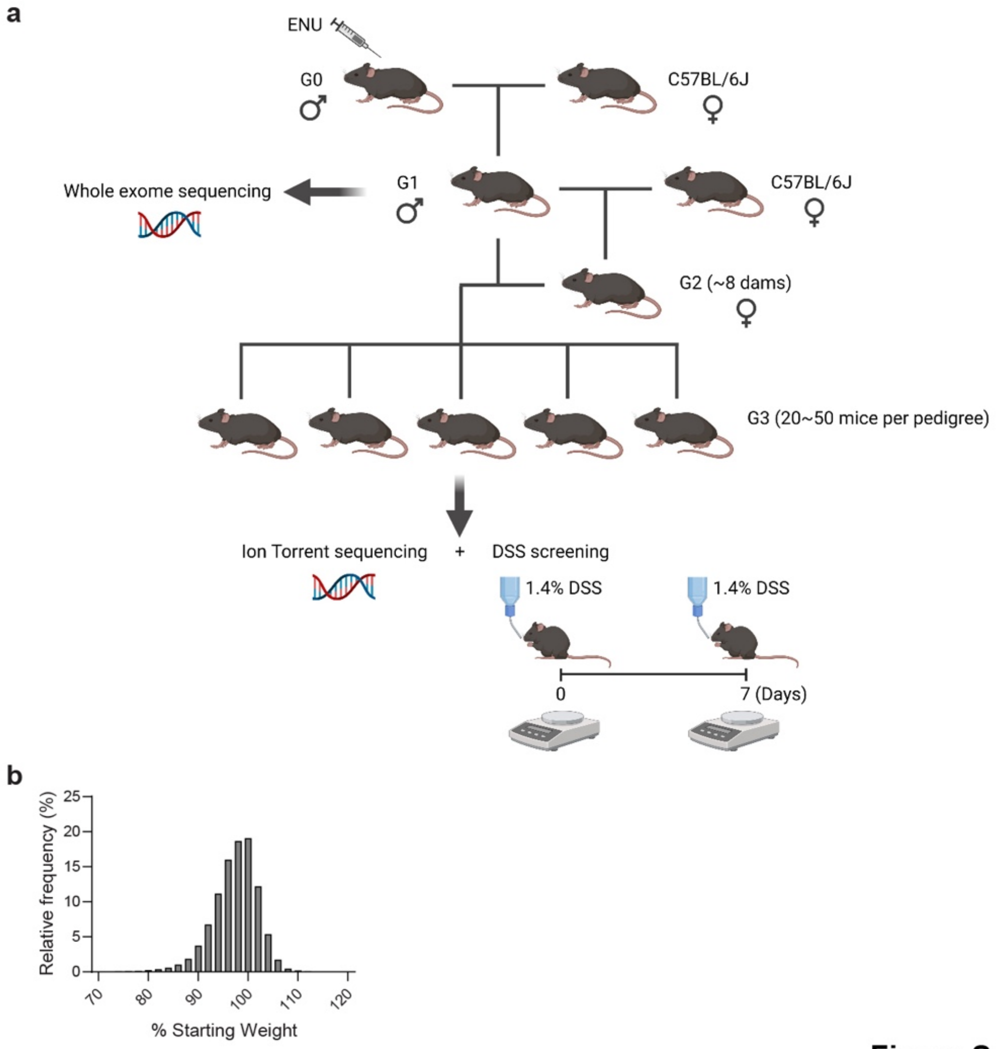

Figure S1

Supplemental Figure S1. Colitis Screening Scheme of ENU-mutagenized mice. (a) Breeding and screening scheme of mice mutagenized with ENU to generate mice for phenotypic screening. First generation (G1) mice define the pedigree and undergo whole exome sequencing to determine ENU induced mutations. Third generation (G3) mice are genotyped according to their G1 grandsire for mutations and then screened for phenotype. G3 mice are for susceptibility to low dose dextran sodium sulfate, measuring weights on Day 0 and Day 7. (b) Distribution of relative weight loss across 55,863 G3 mice tested. Figure S1A was generated in BioRender.

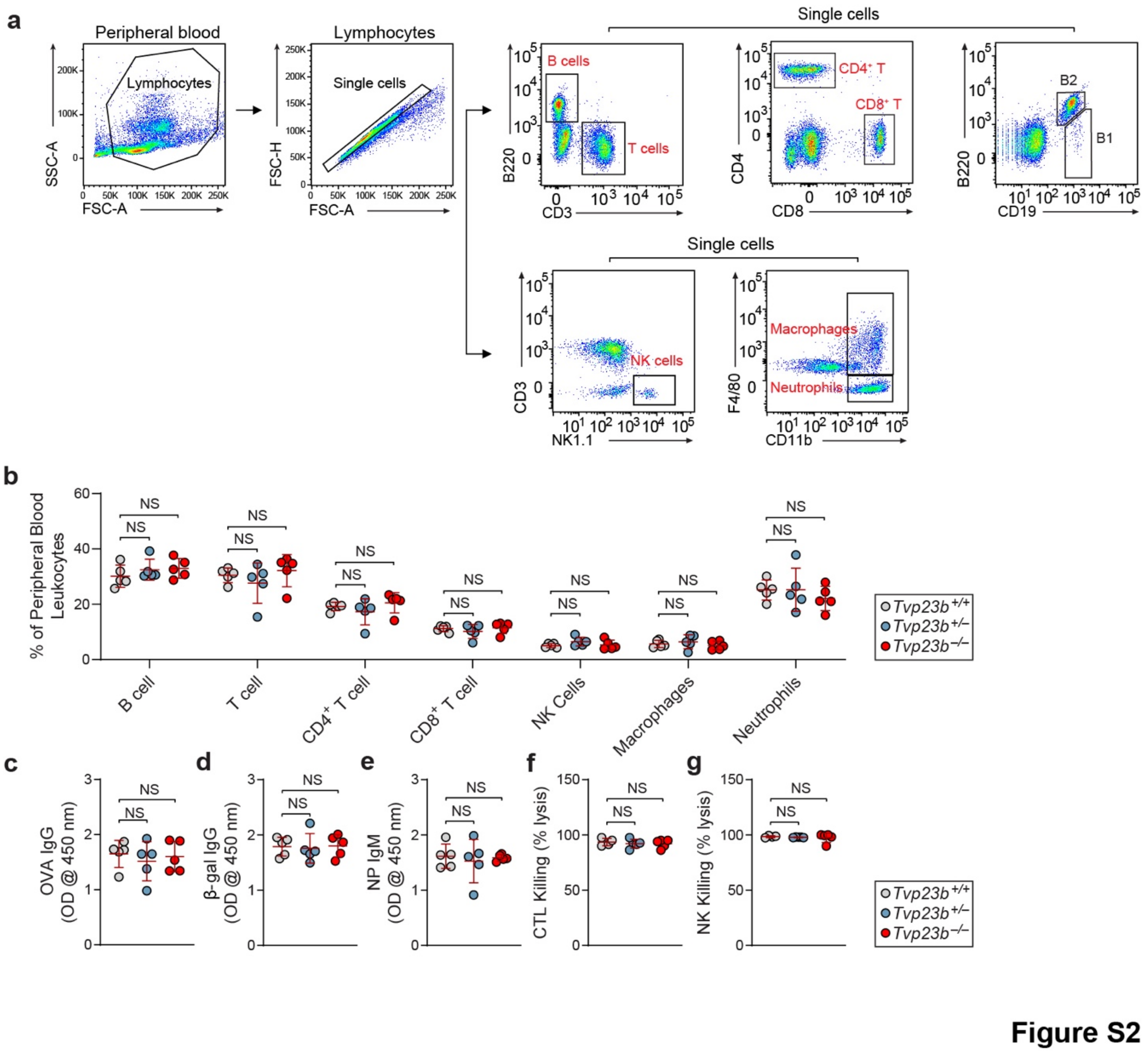

Supplemental Figure S2. Normal peripheral immune homeostasis and responses in TVP23B deficient mice. (a) Gating strategy of peripheral blood leukocytes. (b) Peripheral blood flow cytometry analysis of B cells ($CD19^+$ $B220^+$), T cells ($CD3^+$), $CD4^+$ T cells, $CD8^+$ T cells, macrophages ($CD11b^+$ $F4/80^+$), neutrophils ($CD11b^+$ $F4/80^-$), NK cells ($NK1.1^+$ $CD3^-$) in *Tvp23b*$^{+/+}$, *Tvp23b*$^{+/-}$, and *Tvp23b*$^{-/-}$ mice. (c-e) Serum Ova-specific IgG, b-gal specific IgG and NP-specific IgM measured by ELISA after immunization with Ova/alum, rSFV-b-gal or NP-Ficoll, respectively. (f) $CD8^+$ T cell cytotoxicity was measured against OVA pulsed splenocytes after immunization with Ova/alum. (g) and NK cell cytotoxicity against MHC class I deficient target cells. (N=5 mice/genotype for all experiments). Differences between genotypes were not found to be statistically significant by one way ANOVA. Data are expressed as means ±s.d. ns, not significant.

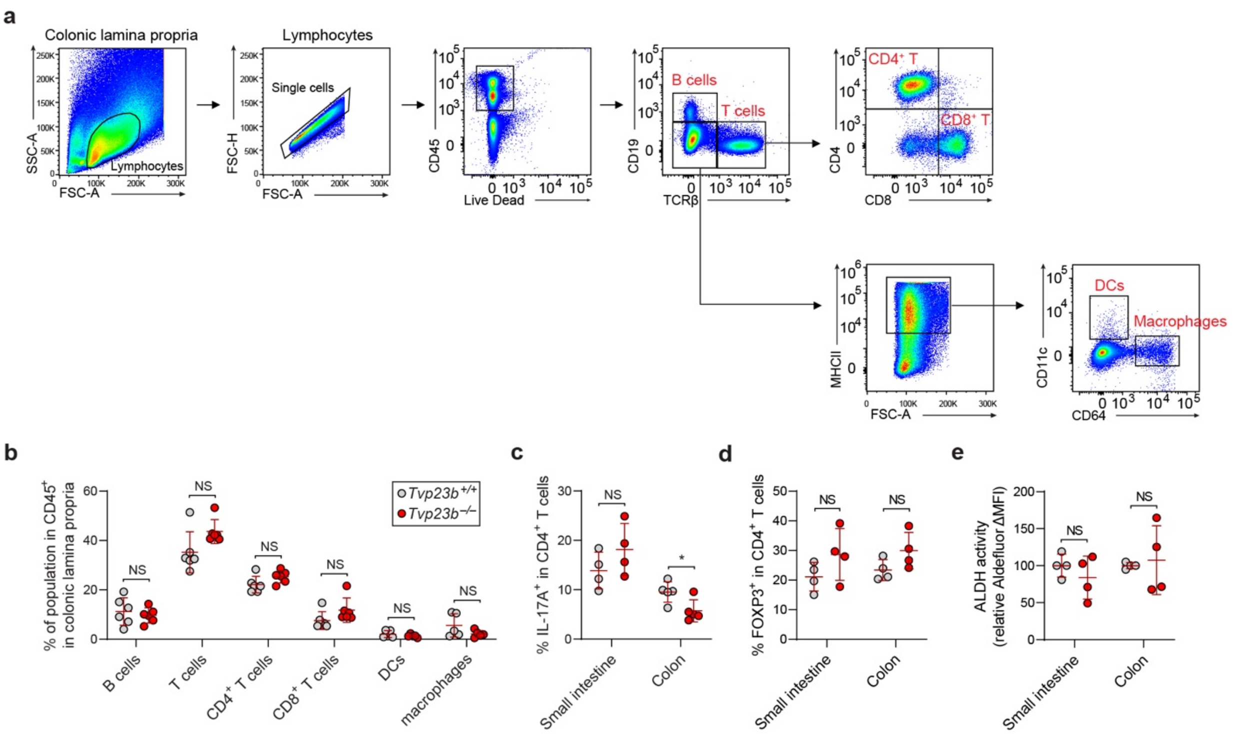

Figure S3

Supplemental Figure S3. Normal intestinal immune homeostasis and responses in TVP23B deficient mice. (a) Gating strategy of lamina propria leukocytes from colons. (b) Colonic lamina propria flow cytometry analysis of B cells ($CD19^+$), T cells ($TCR\beta^+$), $CD4^+$ T cells ($TCR\beta^+$ $CD4^+$), CD8+ T cells ($TCR\beta^+$ $CD8^+$), macrophages ($CD64^+$ $MHCII^+$), Dendritic Cells ($CD11c^+$ $MHCII^+$ $CD64^-$) in *Tvp23b*$^{+/+}$ and *Tvp23b*$^{-/-}$ mice. (n=5 independent mice per genotype) (c) IL17A expression analysis in $CD4^+$ T cells from the small intestines and colons. (d) FOXP3 expression in small intestinal and colonic $CD4^+$ T cells ($TCR\beta^+$ $CD4^+$). (e) ALDH activity as determined by Aldefluor assay of dendritic cells ($CD11c^+$ $MHCII^+$ $CD64^-$) in the small intestines and colons of *Tvp23b*$^{+/+}$ and *Tvp23b*$^{-/-}$ mice. Each data point represents an independent mouse for indicated genotypes. Data are expressed as means ±s.d. and significance was determined by one-way ANOVA (b-e) (*P=0.0239). ns, not significant. Data is reflective of at least 3 independent experiments.

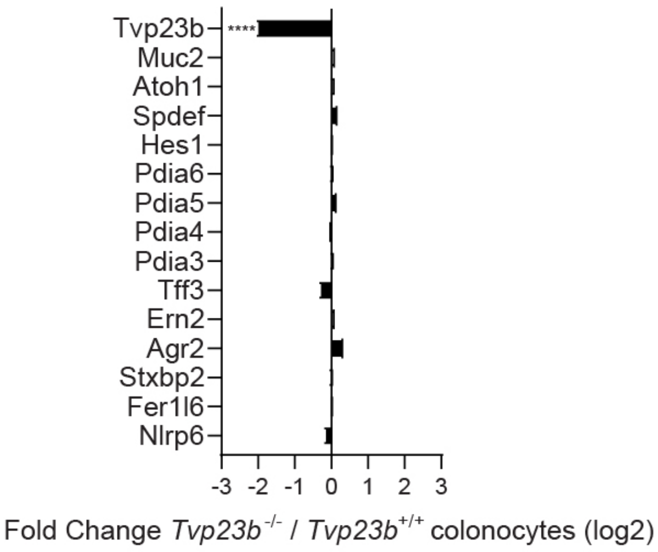

Figure S4

Supplemental Figure S4. Normal RNA-seq Expression of Goblet cell markers. Colonocytes were isolated from the distal colon of *Tvp23b*$^{+/+}$ and *Tvp23b*$^{-/-}$ mice and mRNA was purified from these cells. A total of 3 mice were analyzed per genotype.

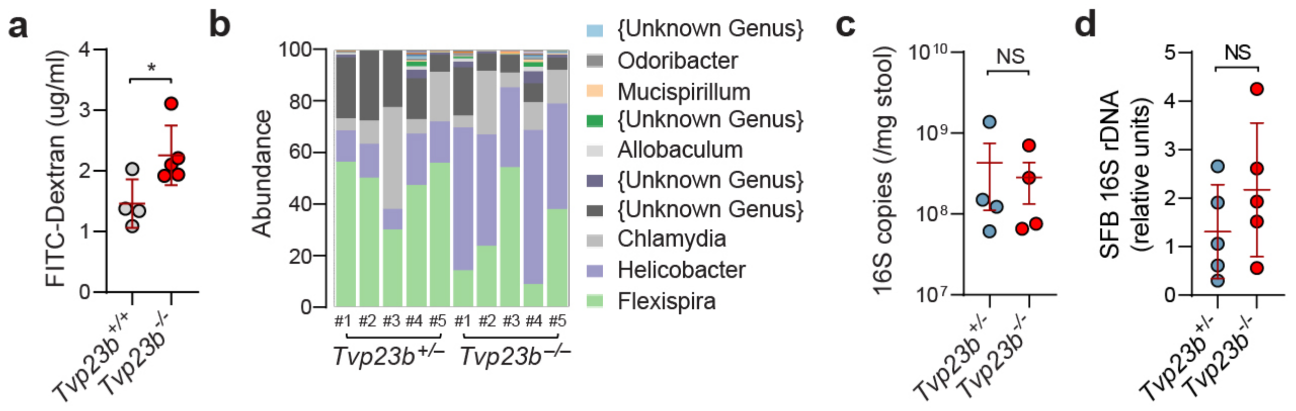

Figure S5

Supplemental Figure S5. Perturbed Intestinal Permeability and microbial colonization. (a) Permeability of the intestine was assessed 4 hours after gavage of mice the impermeable dye FITC dextran (n=4 individual mice, representative of 3 independent experiments, *P=0.035). (b) 16S sequencing of tissue associated bacteria from 5 mice per genotype. Mice were derived from 4 different dams. Each experimental mouse had a corresponding cage/littermate control. (c) Quantitative PCR of eubacteria from fecal samples. (d). Relative segmented filamentous bacteria levels in the small intestine of *Tvp23b*$^{-/-}$ and littermate controls. Number of mice indicated on figures. Data are expressed as means ±s.d. and significance was determined by unpaired Student t-test (a, c, d). *P < 0.05; ns, not significant.

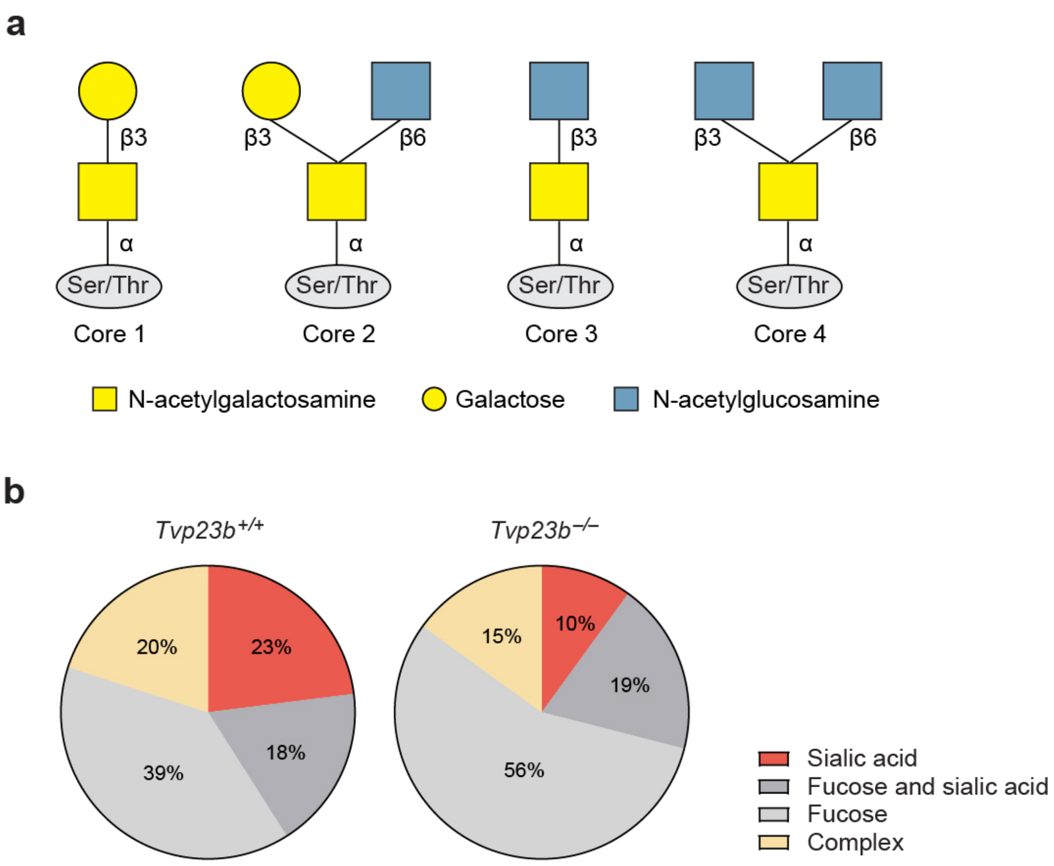

Figure S6

Supplemental Figure S6. Glycosylation in *Tvp23b*$^{-/-}$ colonocytes. (a) Diagram of o-glycosylation forms. (b) Frequency of sialylation and fucosylation of O-glycans in *Tvp23b*$^{-/-}$ colonocytes. Data is representative of 3 samples per genotype with at least 2 mice per sample.

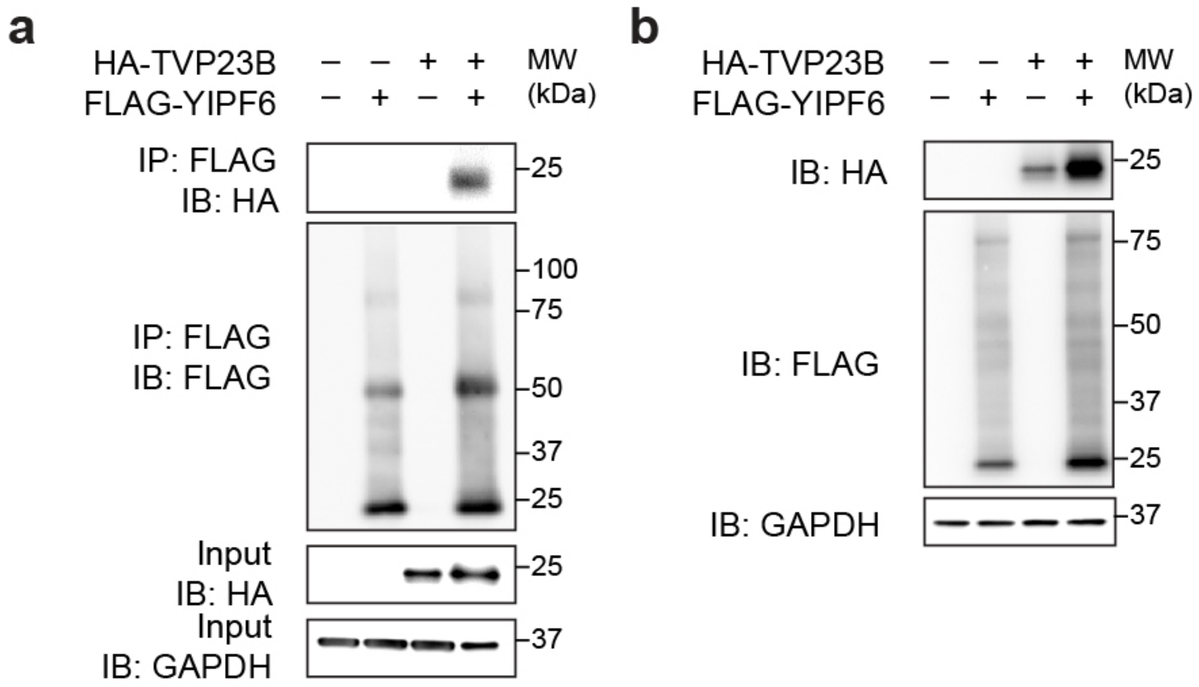

Figure S7

Supplemental Figure S7. Binding and Stabilization of TVP23B by YIPF6. (a) HA-TVP23B and FLAG-YIPF6 were cotransfected into HEK 293T cells and FLAG epitope used for immunoprecipitation. (b) Enhanced TVP23B expression upon cotransfection with FLAG-YIPF6. All data are representative of at least 3 experiments.

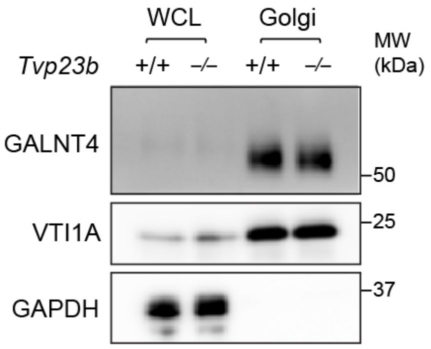

Figure S8

Supplemental Figure S8. Expression of the glycosyltransferase GALNT4. GALNT4 Immunoblot of whole cell lysate and Golgi enriched fraction from *Tvp23b*$^{+/+}$ and *Tvp23b*$^{-/-}$ colonocytes.

Uncropped Blots

Figure 2D

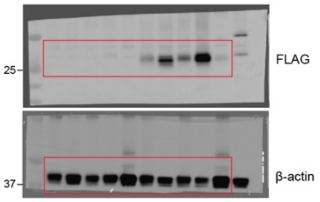

Figure 6D

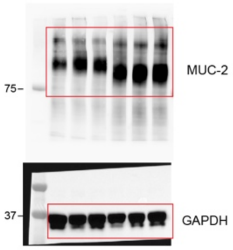

Figure 7A

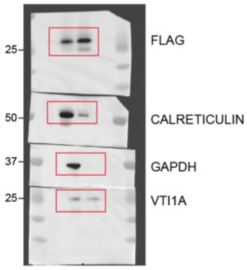

Figure 7B

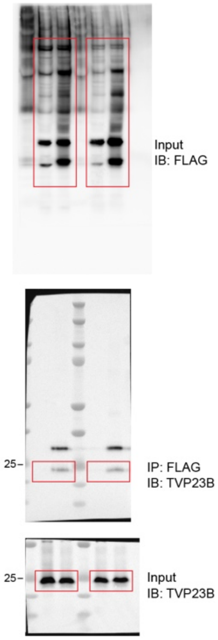

Figure 7C

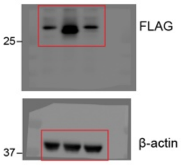

Figure 7G

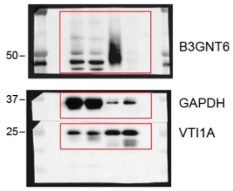

Figure 7H

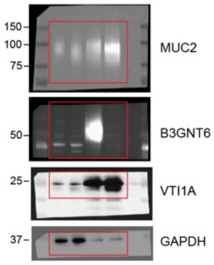

Figure S7A

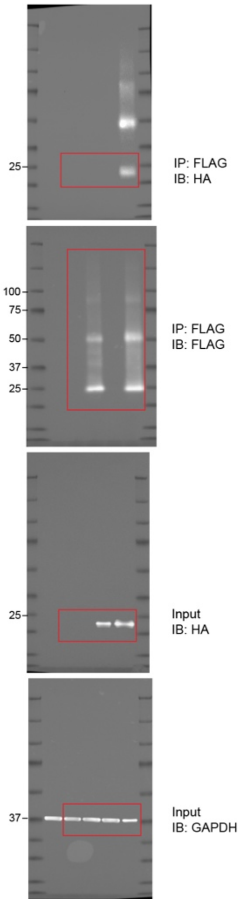

Figure S7B

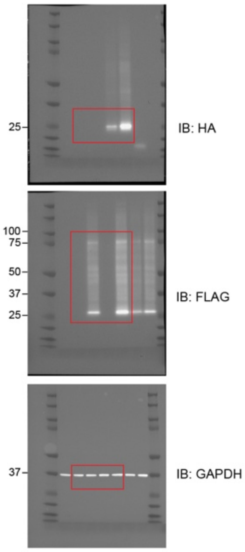

Figure S8

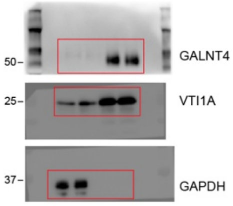

Supplement: Supplementary file 1 — Supplementary Information [file 41467_2023_39398_MOESM1_ESM.pdf]
