# Supplementary material for: Trans-Golgi protein TVP23B regulates host-microbe interactions via Paneth cell homeostasis and Goblet cell glycosylation
Source: Nat Commun. 2023 Jun 20;14:3652. doi: 10.1038/s41467-023-39398-1 (PMC10282085; doi:10.1038/s41467-023-39398-1)
Supplement: Supplementary file 3 — Description of Additional Supplementary Files [file 41467_2023_39398_MOESM3_ESM.pdf]

Description of Additional Supplementary Files

Supplementary Data 1. Raw Proteomic Data of small intestinal peptides. Distal small intestine from *Tvp23b*$^{+/+}$ and *Tvp23b*$^{-/-}$ mice were lysed and analyzed by LC MS/MS.

Supplementary Data 2: Raw O-glycosylation data. Colon epithelial cells were isolated from *Tvp23b*$^{+/+}$ and *Tvp23b*$^{-/-}$ mice were analyzed for O-glycosylation by mass spectroscopy.

Supplementary Data 3: Raw Golgi Proteomics data. Golgi from colonic epithelial cells were purified from *Tvp23b*$^{+/+}$, *Tvp23b*$^{-/-}$, *Yipf6*$^{+/y}$ and *Yipf6*$^{klz/y}$ mice and analyzed by mass spectroscopy.

Supplementary Data 4. Table of Key resources.
