# Supplementary material for: Trans-Golgi protein TVP23B regulates host-microbe interactions via Paneth cell homeostasis and Goblet cell glycosylation
Source: Nat Commun. 2023 Jun 20;14:3652. doi: 10.1038/s41467-023-39398-1 (PMC10282085; doi:10.1038/s41467-023-39398-1)
Supplement: Supplementary file 8 — Reporting Summary [file 41467_2023_39398_MOESM8_ESM.pdf]

nature portfolio

Corresponding author(s): Emre Erol Turer

Last updated by author(s): May 24, 2023

Reporting Summary

Nature Portfolio wishes to improve the reproducibility of the work that we publish. This form provides structure for consistency and transparency in reporting. For further information on Nature Portfolio policies, see our Editorial Policies and the Editorial Policy Checklist.

Statistics

For all statistical analyses, confirm that the following items are present in the figure legend, table legend, main text, or Methods section.

n/a	Confirmed	
☐	☒	The exact sample size (*n*) for each experimental group/condition, given as a discrete number and unit of measurement
☐	☒	A statement on whether measurements were taken from distinct samples or whether the same sample was measured repeatedly
☐	☒	The statistical test(s) used AND whether they are one- or two-sided<br>*Only common tests should be described solely by name; describe more complex techniques in the Methods section.*
☒	☐	A description of all covariates tested
☐	☒	A description of any assumptions or corrections, such as tests of normality and adjustment for multiple comparisons
☐	☒	A full description of the statistical parameters including central tendency (e.g. means) or other basic estimates (e.g. regression coefficient) AND variation (e.g. standard deviation) or associated estimates of uncertainty (e.g. confidence intervals)
☐	☒	For null hypothesis testing, the test statistic (e.g. *F*, *t*, *r*) with confidence intervals, effect sizes, degrees of freedom and *P* value noted<br>*Give P values as exact values whenever suitable.*
☒	☐	For Bayesian analysis, information on the choice of priors and Markov chain Monte Carlo settings
☒	☐	For hierarchical and complex designs, identification of the appropriate level for tests and full reporting of outcomes
☒	☐	Estimates of effect sizes (e.g. Cohen's *d*, Pearson's *r*), indicating how they were calculated

Our web collection on statistics for biologists contains articles on many of the points above.

Software and code

Policy information about availability of computer code

Data collection	FLOW cytometry was acquired on a BD LSRFortessa (BD Biosciences). RNA sequencing was performed using an Illiumina Hiseq 2500.
Data analysis	ImageJ (v 2.9.0) was used to quantify all microscopy.<br>FlowJo (v 10.9) was used for FLOW cytometric analysis<br>For RNAsequencing, CASAVA and tophat (v 2.0.10) and cuffmerge (v 2.1.1) were used.<br>For proteins extracted from ileum, protein and peptide identification were done with ProLucid (version1.4) and DTASelect (v2.1.12).<br>For Golgi apparatus enrichment, protein and peptide identification were done with MSFragger (version 17.1)

For manuscripts utilizing custom algorithms or software that are central to the research but not yet described in published literature, software must be made available to editors and reviewers. We strongly encourage code deposition in a community repository (e.g. GitHub). See the Nature Portfolio guidelines for submitting code & software for further information.

Data

Policy information about availability of data

All manuscripts must include a data availability statement. This statement should provide the following information, where applicable:

- Accession codes, unique identifiers, or web links for publicly available datasets
- A description of any restrictions on data availability
- For clinical datasets or third party data, please ensure that the statement adheres to our policy

All raw proteomic data has been deposited at the ProteomeXchange with source code PXD038751, PXD038895. RNAsequencing data is available with reference GSE224516. Source data are provided with this paper.

Research involving human participants, their data, or biological material

Policy information about studies with human participants or human data. See also policy information about sex, gender (identity/presentation), and sexual orientation and race, ethnicity and racism.

Reporting on sex and gender	Not applicable
Reporting on race, ethnicity, or other socially relevant groupings	Not applicable
Population characteristics	Not applicable
Recruitment	Not applicable
Ethics oversight	Not applicable

Note that full information on the approval of the study protocol must also be provided in the manuscript.

Field-specific reporting

Please select the one below that is the best fit for your research. If you are not sure, read the appropriate sections before making your selection.

☒ Life sciences ☐ Behavioural & social sciences ☐ Ecological, evolutionary & environmental sciences

For a reference copy of the document with all sections, see nature.com/documents/nr-reporting-summary-flat.pdf

Life sciences study design

All studies must disclose on these points even when the disclosure is negative.

Sample size	No sample size calculation was performed. In general at least 4 mice were used. This sample size was sufficient to demonstrate statistically significant differences in comparisons between two unpaired experimental groups by unpaired t-test or ANOVA, where indicated
Data exclusions	No data was excluded
Replication	RNAsequencing of colonic epithelium was performed once. All other experiments were repeated at least three times and all replicate attempts were successful.
Randomization	Mice were not randomized to ensure adequate numbers of each genotype per condition.
Blinding	Investigators were not blind to the number of experimental mice in each group to ensure adequate numbers of genotypes per condition.

Reporting for specific materials, systems and methods

We require information from authors about some types of materials, experimental systems and methods used in many studies. Here, indicate whether each material, system or method listed is relevant to your study. If you are not sure if a list item applies to your research, read the appropriate section before selecting a response.

Materials & experimental systems

n/a	Involved in the study
☐	☒ Antibodies
☐	☒ Eukaryotic cell lines
☒	☐ Palaeontology and archaeology
☐	☒ Animals and other organisms
☒	☐ Clinical data
☒	☐ Dual use research of concern
☒	☐ Plants

Methods

n/a	Involved in the study
☒	☐ ChIP-seq
☐	☒ Flow cytometry
☒	☐ MRI-based neuroimaging

Antibodies

Antibodies used

All antibody dilutions noted in Key reagent table
Anti-mouse B220 (AF700); clone RA3-6B2 BD Biosciences Cat# 557957; RRID:AB_396957
Anti-mouse CD19 (BUV395); clone 1D3 BD Biosciences Cat# 563557; RRID:AB_2722495
Anti-mouse/human CD11b (BV605); clone M1/70 Biolegend Cat# 101257; RRID:AB_2565431
Anti-mouse CD3ε (FITC); clone 145-2C11 Biolegend Cat# 100306; RRID:AB_312671
Anti-mouse CD4 (BV786); clone RM4-5 BD Biosciences Cat# 563727; RRID:AB_2728707
Anti-mouse CD8α (BV510); clone 53-6.7 Biolegend Cat# 100752; RRID:AB_2563057
Anti-mouse F4/80 (PE); clone BM8.1 TONBO Cat# 50-4801; RRID:AB_2621795
Anti-mouse NK1.1 (BV650); clone PK136 BD Biosciences Cat# 564143; RRID:AB_2738617
Anti-mouse CD45 (BV395); clone 30-F11 BD Biosciences Cat# 564279; RRID:AB_2651134
Anti-mouse TCRβ (BV421); clone H57-597 Biolegend Cat# 109230; RRID:AB_2562562
Anti-mouse CD11c (BV711); clone HL3 BD Biosciences Cat# 564279; RRID:AB_2734778
Anti-mouse CD64 (PE/Cyanine7); clone X54-5/7.1 Biolegend Cat# 139314; RRID:AB_2563904
Anti-mouse MHC-II (PE); clone M5/114.15.2 Thermo Fisher Scientific Cat# 12-5321-82; RRID:AB_465928
Anti-mouse Foxp3 (Alexa Fluor 647); clone MF23 BD Biosciences Cat# 560401; RRID:AB_1645201
Anti-mouse IL-17A (PE); clone TC11-18H10 BD Biosciences Cat# 559502; RRID:AB_397256
GAPDH (D16H11) XP Rabbit mAb Cell Signaling Technologies Cat# 5174; RRID:AB_10622025
B3GALT6 Polyclonal antibody Proteintech Cat# 55049-1-AP; RRID:AB_10888634
GALNT4 Polyclonal antibody Proteintech Cat# 12897-1-AP; RRID:AB_10643389
Recombinant Anti-MUC2 antibody Abcam Cat# ab272692; RRID:AB_2888616
DYKDDDDK tag Polyclonal antibody Proteintech Cat# 20543-1-AP; RRID:AB_11232216
Anti-Calreticulin antibody Abcam Cat# ab92516; RRID:AB_10562796
Purified Mouse Anti-Vti1a BD Biosciences Cat# 611220; RRID:AB_398752
FAM18B Polyclonal Antibody Thermo Fisher Scientific Cat# PA5-54030; RRID:AB_2641352
Anti-rabbit secondary antibody Cell Signaling Technology Cat# 7074; RRID:AB_2099233
Anti-mouse secondary antibody Cell Signaling Technology Cat# 7076; RRID:AB_330924

Validation

All primary antibodies used in this study were commercially purchased and validation was performed by the individual companies. Validation data for the specific application is present on the data sheets provided by the company websites.

Eukaryotic cell lines

Policy information about cell lines and Sex and Gender in Research

Cell line source(s)

LentiX 293T (Takara)
HT29-MTX (Sigma)
LS174T (ATCC)

Authentication

All cell lines were commercially purchased and the manufacturer tested and authenticated these cells, including morphological testing

Mycoplasma contamination

All cell lines were periodically tested for mycoplasma and all tested negative

Commonly misidentified lines
(See ICLAC register)

none used in this study

Animals and other research organisms

Policy information about studies involving animals; ARRIVE guidelines recommended for reporting animal research, and Sex and Gender in Research

Laboratory animals

Pure C57BL6/J mice were used for these studies. All mice were 8-12 weeks of age

Wild animals

none used in this study

Reporting on sex	Both male and female mice were used in this study. No gender bias was found.
Field-collected samples	no field collected samples
Ethics oversight	All experiments were performed under and conducted in accordance with an animal protocol approved by the UT Southwestern Medical Center IACUC office.

Note that full information on the approval of the study protocol must also be provided in the manuscript.

Flow Cytometry

Plots

Confirm that:

- [ ] The axis labels state the marker and fluorochrome used (e.g. CD4-FITC).
- [x] The axis scales are clearly visible. Include numbers along axes only for bottom left plot of group (a 'group' is an analysis of identical markers).
- [x] All plots are contour plots with outliers or pseudocolor plots.
- [x] A numerical value for number of cells or percentage (with statistics) is provided.

Methodology

Sample preparation	As in methods section. PBL samples wer collected in heparin coated tubes. RBCs lysed and washed three times prior to antibody staining. Lamina propria cells were isolated from digested tissues. leukocytes were further isolated by percoll gradient prior to antibody staining
Instrument	LSR Fortessa (BD Biosciences)
Software	FlowJo was used for all analysis work.
Cell population abundance	All cell populations were abundant, in a reasonable range, and significant to gate
Gating strategy	For peripheral blood and lamina propria leukocytes, all gating strategies are included in the supplemental figure section. Briefly, cells were gated on forward scatter and side scatter. Single cells were then gated as well live cells by use of a viability dye.

- [x] Tick this box to confirm that a figure exemplifying the gating strategy is provided in the Supplementary Information.
